# Activation and Biological Properties of Human β Defensin 4 in Stem Cells Derived From Human Exfoliated Deciduous Teeth

**DOI:** 10.3389/fphys.2019.01304

**Published:** 2019-10-22

**Authors:** Yue Zhai, Yuanyuan Wang, Nanquan Rao, Jingzhi Li, Xiaoxia Li, Tengjiaozi Fang, Yuming Zhao, Lihong Ge

**Affiliations:** Department of Pediatric Dentistry, Peking University School and Hospital of Stomatology, Beijing, China

**Keywords:** human β defensin 4, SHED, anti-inflammatory, stem cell differentiation, pulp regeneration

## Abstract

Pulpitis in primary teeth, a condition caused by presence of bacteria, is highly prevalent worldwide. The use of biocompatibility materials with anti-inflammatory, anti-bacterial, and regenerative properties is critical for prognosis of this endodontic disease. This study aimed to identify expression of human β defensin 4 (HBD4) in stem cells derived from human exfoliated deciduous teeth (SHED) and characterize the effects of HBD4 on SHED. Quantitative polymerase chain reaction (qPCR) was used to detect HBD4 expression in SHED and the effect of HBD4 on inflammatory factors in lipopolysaccharide (LPS)-stimulated SHED. Affinity measurement was made by the Fortebio Octet System to explore the potential interaction between LPS and HBD4. Western blot analysis was used to explore the effect of HBD4 on mitogen-activated protein kinase (MAPK) pathway. Colony-forming unit methods and scanning electron microscopy were applied to study antimicrobial effect of HBD4 on *Fusobacterium nucleatum* and *Porphyromonas gingivalis*. Alkaline phosphatase staining, alizarin red staining, qPCR and western blot were taken to detect effects of HBD4 on osteoblast/odontoblast differentiation of SHED. RT^2^ Profiler PCR Array was used to explore the potential signaling pathways involved in the osteogenic/odontogenic differentiation. HBD4 was highly expressed in SHED stimulated by TNF-α and IL-1α. HBD4 could bind to LPS directly and down-regulate IL-1α, IL-1β, IL-6, TNF-α in LPS-stimulated SHED, thus the activation of MAPK pathway decreased. HBD4 was sensitive to *P. gingivalis* and enhanced osteoblast/odontoblast differentiation potential of SHED by modulating Notch pathway. HBD4 was highly expressed in SHED stimulated by proinflammatory cytokines, and possessed anti-inflammatory, anti-bacterial activity. HBD4 promoted osteogenic/odontogenic differentiation of SHED. HBD4 may thus represent a suitable agent for vital pulp therapy in future clinic application.

## Introduction

Pulpitis is one of the most common diseases of primary teeth. Pulpitis of primary teeth can induce a series of physiological and pathological changes such as local inflammation, edema and congestion of pulp tissue. It may also cause serious harm to the development of permanent tooth germ in children. Dental pulps with pulpitis suffer higher expressions of proinflammatory cytokines (IL-1α, IL-1β, IL-6, and TNF-α) and innate immune response (TLR2, TLR4) than pulps without pulpitis. It is now well known that the key to successful pulp therapy is to reduce the degree of pulpal inflammation (Ozdemir et al., 2015; Tsai et al., 2017; Mutluay et al., 2018). Vital pulp therapy is a minimal intervention strategy to maximize the preservation of living pulp tissues and avoid root canal therapy or tooth extraction. This procedure involves the application of pulp capping agent on a nearly exposed pulp (indirect capping), an exposed coronal pulp (direct capping), or removed coronal pulp tissue (pulpotomy) to form new dentin-pulp complex (da Rosa et al., 2018). Existing pulp capping agents cannot retain pulp tissue with irreversible inflammation, but can preserve the dental pulp in the case of reversible pulpitis. Calcium hydroxide has a long history as pulp capping material in the clinic. However, the ability of calcium hydroxide to resist anaerobic bacteria is weak; the strong alkalinity and cytotoxicity of calcium hydroxide can lead to coagulative necrosis of superficial dental pulp tissue (Schwendicke et al., 2016). Currently, mineral trioxide aggregate (MTA) is identified as the best treatment option for pulpotomy in primary teeth. MTA has good biocompatibility, dentinogenic and osteogenic potential. In addition, it is effective in controlling pulp inflammation (Nair et al., 2008). However, MTA has drawbacks such as discoloration, the presence of toxic elements (arsenic), and higher cytotoxicity in its freshly mixed state (Balto, 2004; Bogen et al., 2008).

The request to find an ideal pulp capping agent has led to the research of bioactive protein, as bioactive protein may potentiate the natural pulp healing process and stimulate the biological tissue(Luiz de Oliveira da Rosa et al., 2017). The natural repair and regenerative process in the dentin-pulp complex of primary teeth has been extensively studied by researchers. One possible mechanism is that stem cells contained in the pulp tissue are mobilized when the pulp of the primary tooth is injured. Damaged odontoblasts are replaced by odontoblasts derived from stem cells differentiation, and then new dentin matrix is formed. Therefore, the key point is to develop bioactive protein that can activate stem cells contained in pulp tissue and provide good differentiation microenvironment.

Human β defensin (HBD) is a family of small cationic polypeptides rich in cysteine. Thus far, more than 50 HBD genes have been discovered (Pazgier et al., 2006) and HBD1-4 are the most frequently studied members of the HBD family. HBD has a direct effect on most bacteria (Joly et al., 2004), some fungi and viruses (Schneider et al., 2005; Wilson et al., 2013). The common way it acts on microorganisms is through its own positive charges to interact with negatively charged components of bacterial cell membranes, such as lipopolysaccharide (LPS). Thus, the permeability of bacterial cell membranes is increased, and bacterial death is caused by bacterial lysis (Matsuzaki, 1999). In addition to its antimicrobial effect, HBD has important functions such as inflammation inhibitory effect (Semple et al., 2010; Liu H. et al., 2013), regulation of differentiation (Kraus et al., 2012), and anti-cancer effect (Uraki et al., 2014). Currently, the studies of HBD in dental pulp focus on pulp tissue and pulp cells from permanent teeth. Dommisch et al. (2005) first reported the expression of HBD in dental pulp tissue in 2005. Some stimuli associated with inflammatory immune response in dental pulp cells can increase the expression of HBD2 (Kim et al., 2010; Lee et al., 2011, 2015). Adding HBD2 at the late stage of odontoblast induction could increase the expression of dentin sialophosphoprotein messenger RNA in human dental pulp cells (Shiba et al., 2003). Nevertheless, the expression and function of HBD in pulp tissue and pulp cells from primary teeth are rarely reported.

HBD is a type of endogenous polypeptide with biocompatibility. In addition, HBD has good anti-inflammatory and bacteriostatic abilities, as well as the potential to promote the differentiation of stem cells into odontoblasts (Shiba et al., 2003) or osteoblasts (Kraus et al., 2012). These properties of HBD are necessary for ideal pulp capping agents. As our preliminary studies showed that HBD4 was highly expressed in stem cells from human exfoliated deciduous teeth (SHED), we used HBD4 as a point of reference to study the relationship between HBD and SHED. The current research will be helpful to discover a new ingredient of pulp capping agent for primary teeth.

Therefore, the objectives of this *in vitro* study were to: (1) evaluate the expression level of HBD4 in SHED; (2) figure out the ability of HBD4 in inhibiting LPS-mediated inflammation in SHED; (3) test the antibacterial activity of HBD4 against pathogenic bacteria of pulpitis; and (4) explore the ability of HBD4 in inducing SHED to differentiate into odontoblasts and osteoblasts.

## Materials and Methods

### Cell Culture and Markers Profile Expression

Exfoliated deciduous teeth were collected from healthy patients aged 6–8 years (*n* = 3) from the Department of Pediatric Dentistry, Peking University School and Hospital of Stomatology. Totally three different teeth were collected and three different SHED primary cell lines were generated and examined following the exact same procedures and methods. Results obtained from the three different SHED cultures were similar. The research was approved by the Ethics Committee of the Peking University Health Science Center (PKUSSIRB-201630091). SHED were isolated from exfoliated deciduous teeth according to the protocol developed by Miura et al. (2003). Cells were then cultured in α-minimum essential medium (Hyclone, Logan, UT, United States) supplemented with 10% fetal bovine serum (FBS, Gibco, Mulgrave, VIC, Australia), 100 U/mL penicillin, and 100 μg/mL streptomycin (Solarbio, Beijing, China) in a humidified atmosphere of 5% CO_2_ at 37°C. The cells used in experiments were between passage 3 and 5.

The surface markers of SHED were identified by flow cytometry. Cells were detached with trypsin/ethylenediaminetetraacetic acid (Gibco) to produce single-cell suspension and were resuspended in phosphate buffered saline (PBS) containing 2% FBS. Cells at a concentration of 1 × 10^6^ cells/mL were then added with the following monoclonal antibodies: CD34-PE, CD45-PE, CD73-PE, CD90-FITC, CD105-FITC and CD146-PE (BD Pharmingen, San Diego, CA, United States). The stained cells were analyzed using the flow cytometry system (FC500, Beckman Coulter, Brea, CA, United States) to detect fluorescence intensity and positive rate.

### Quantitative Polymerase Chain Reaction

RNA was isolated using TRIzol (Invitrogen, Carlsbad, CA, United States) following the manufacturer’s instructions. Reverse transcription of RNA was performed using transcriptor first-strand complementary DNA synthesis kit (Takara Biotechnology, Dalian, Liaoning, China). The primers used in quantitative polymerase chain reaction (qPCR) were outlined in Table 1. The qPCR with total RNA was performed with a SYBR Green System (7300 Real Time System, Applied Biosystems, Carlsbad, CA, United States) according to the manufacturer’s protocol. The target gene expressions were normalized with β-actin. Relative gene expression values were calculated by ΔΔCT-based fold-change calculations. The qPCR products of HBD1-4 were resolved on a 1.5% agarose gel and stained with SYBR Green I (Solarbio).

**TABLE 1:** Primers used for qPCR.

**Target gene**	**Sequence**	**Product size (bp)**	**GenBank number**
β-actin	Forward: CCTGGCACCCAGCACAAT	144	NM_001101.5
	Reverse: GGGCCGGACTCGTCATACT		
HBD1	Forward: CATGAGAACTTCCTACCTTCTGC	208	NM_005218.4
	Reverse: TCACTTGCAGCACTTGGCCTT		
HBD2	Forward: ATCAGCCATGAGGGTCTTGT	172	AF040153
	Reverse: GAGACCACAGGTGCCAATTT		
HBD3	Forward: AGCCTAGCAGCTATGAGGATC	206	NM_018661.4
	Reverse: CTTCGGCAGCATTTTCGGCCA		
HBD4	Forward: TGCCTTAAGAGTGGAGCCATA	109	NM_004942
	Reverse: CTCCTCATGGCTTTTTGCAG		
TNF-α	Forward: GGCTCCAGGCGGTGCTTGTTC	190	NM_000594.4
	Reverse: CAGGCTTGTCACTCGGGGTTCG		
IL-6	Forward: GGTGTTGCCTGCTGCCTTCC	193	NM_000600.5
	Reverse: TGCCTCTTTGCTGCTTTCACAC		
IL-1α	Forward: TGAAGGCAAAGCACGAAATGTTAT	198	NM_000575.4
	Reverse: TGGACCAAAATGCCCTGTAT		
IL-1β	Forward: GGCAGGCCGCGTCAGTTG	198	NM_000576.2
	Reverse: CCCGGAGCGTGCAGTTCAGT		
TLR2	Forward: GCGTGGCCAGCAGGTTCAGG	167	XM_011532216.2
	Reverse: GGAGCCAGGCCCACATCATTTTC		
TLR4	Forward: TGCAATGGATCAAGGACCAG	147	NM_138554.5
	Reverse: TGAGGACCGACACACCAATG		
Runx-2	Forward: CTGAGGTAACTTGCTAACG	101	NM_001024630.4
	Reverse: ATCAATACACTAAGAAATGTTTCAAGG		
OCN	Forward: AGCAAAGGTGCAGCCTTTGT	261	NM_199173.5
	Reverse: GCGCCTGGGTCTCTTCACT		
DMP-1	Forward: TGGGGATTATCCTGTGCTCT	129	XM_011531706.2
	Reverse: GCTGTCACTGGGGTCTTCAT		
DSPP	Forward: TCCTAGCAAGATCAAATGTGTCAGT	152	NM_014208.3
	Reverse: CATGCACCAGGACACCACTT		

### Cell Viability Assay and *Trans*-Well Migration of SHED

Cell viability assays were performed using Cell Counting Kit-8 (Beyotime, Shanghai, China). SHED at a concentration of 5 × 10^4^ cells/mL were plated in 96-well plates. After overnight incubation, the cells were treated with HBD4 (Beyotime) at the indicated times. A total of 10 μL CCK-8 solution was then added to each well. After incubation for 1 h at 37°C, the optical density (OD) value of each well was measured at a wavelength of 450 nm. The experiments were performed in triplicate.

SHED were cultured in serum free medium overnight. A total of 100 μL 5 × 10^4^ cells were seeded in the upper chamber of a *trans*-well culture plate (8 μm pore size, 24-well) (Corning, Corning, CA, United States). Four groups were set in the bottom chamber: control; 10% FBS; 10 μg/mL HBD4, and 20 μg/mL HBD4. Cells were incubated for 24 h, fixed with methanol, and stained with Giemsa Stain solution (Solarbio, Beijing, China). The unmigrated cells were wiped from the bottom of the upper chamber with a wet cotton stick. The number of migrated cells were counted using a microscope. Three random visual fields were counted and the average was taken as the number of migrated cells per group. The experiments were performed in triplicate.

### Affinity Measurements Between LPS and HBD4

HBD4 was dialyzed in PBS and biotinylated at room temperature for 30 min. The biotin-conjugated HBD4 was diluted to 50 μg/mL and 25 μg/mL. The sensors (Streptavidin) were prewet in PBS for 15 min before use. The sensors loading with PBS were used as the control group. LPS (Sigma-Aldrich, St. Louis, MO, United States) was prepared at 1 mg/mL in a 96-well plate. The measurements were carried out by the Fortebio Octet System at room temperature.

### Western Blot

The cell samples were harvested and washed with PBS and subsequently lysed in RIPA lysis buffer with phenylmethylsulphfonyl fluoride (Beyotime). The protein concentrations were detected using Pierce BCA Protein Assay Kit (Thermo Fisher Scientific, Waltham, MA, United States). The protein samples were separated on sodium dodecyl sulfatesulphate polyacrylamide gel and transferred to polyvinylidene difluoride membranes. The membranes were incubated with primary antibodies at 4°C overnight against Runx2(1:1000), p42/44 mitogen-activated protein kinase (MAPK; 1:1000), Phospho-p42/44 MAPK (1:2000), hairy and enhancer of split-1 (HES1; 1:1000), β-actin (1:10000) (all from Cell Signaling Technology, Beverly, MA, United States), Dentin sialophosphoprotein (DSPP; 1:500) and Dentin matrix acidic phosphoprotein 1 (DMP-1; 1:500) (both from Santa Cruz Biotechnology, Santa Cruz, CA, United States). Subsequently, the membranes were incubated with corresponding secondary antibodies for 1 h. The blotted bands were detected using ECL Western Blotting Substrate (Solarbio).

### *In vitro* Antibacterial Activity of HBD4

*Fusobacterium nucleatum* (ATCC 25586) and *Porphyromonas gingivalis* (ATCC 33277) were used in the antibacterial activity research. These strains were cultured in brain-heart infusion media. The anaerobic culture conditions were 80% N_2_, 10% H_2_, and 10% CO_2_ at 37°C. Overnight cultures of bacterial strains were harvested, and the bacterial suspension was diluted to 10^6^ cells/mL with H_2_O. A total of 4 μL bacterial suspension was mixed with 36 μL HBD4 (diluted in H_2_O at the concentrations of 500, 250, 125, 62.5, 31.2, 15.6 μg/mL) and the mixture was incubated in anaerobic culture conditions for 3 h at 37°C. The reaction mixtures were plated on blood agar medium and colony counts were performed after 48 h of incubation at 37°C. The antibacterial effect was estimated as the ratio between surviving cells and total cells. The experiments were performed for three times.

Next, 1 × 10^5^ cells/mL *P. gingivalis* were incubated with 100 μg/mL HBD4 or H_2_O at 37°C for 3 h and then the two reaction mixtures were observed by scanning electron microscopy (SEM) separately. Briefly, the reaction mixture was washed with PBS, fixed with 4% paraformaldehyde, serially dehydrated, dried, and observed with JEOL JSM-7900F SEM.

### Alkaline Phosphatase Staining and Alizarin Red Staining

The cells were cultured in four groups: mineralization induction, mineralization induction +10 μg/mL HBD4, mineralization induction +1 μg/mL LPS, mineralization induction +1 μg/mL LPS +10 μg/mL HBD4. The concentration of LPS used in this study referred to the previous data in our laboratory (Wang et al., 2019). Alkaline phosphatase (ALP) activities of SHED in the different groups were measured with Alkaline Phosphatase Assay Kit (Beyotime) following the manufacturer’s protocol after 7 days incubation. The quantitative ALP activity was determined by measuring the OD values at 405 nm after incubation with *p*-nitrophenyl phosphate (Beyotime). After culturing for 21 days, the cells were fixed and stained with 2% Alizarin Red S (ARS) (Sigma-Aldrich) solution. The photographs were captured. The stained cells were then destained with 10% acetylpyridinium chloride (Sigma-Aldrich) and measured for OD values at 590 nm.

### Human Signal Transduction PathwayFinder RT^2^ Profiler PCR Array

The Human Signal Transduction PathwayFinder RT^2^ Profiler PCR Array (Qiagen, Dusseldorf, Germany) was applied according to the manufacturer’s protocols to determine the potential signaling pathway related to HBD4 differentiation-promoting effect. Data were analyzed automatically online by Qiagen data analysis tool.

### Statistical Analysis

The statistics were analyzed by SPSS 17.0 (SPSS Inc., Chicago, IL, United States). Data were presented as the mean ± standard deviation (SD). Comparisons between two groups were analyzed using the *t*-test. One-way analysis of variance was applied for multiple comparisons. A value of *P* ≤ 0.05 was considered significant.

## Results

### Activated Expression of HBD and Effects of HBD4 on Biological Activity of SHED

**FIGURE 1:**
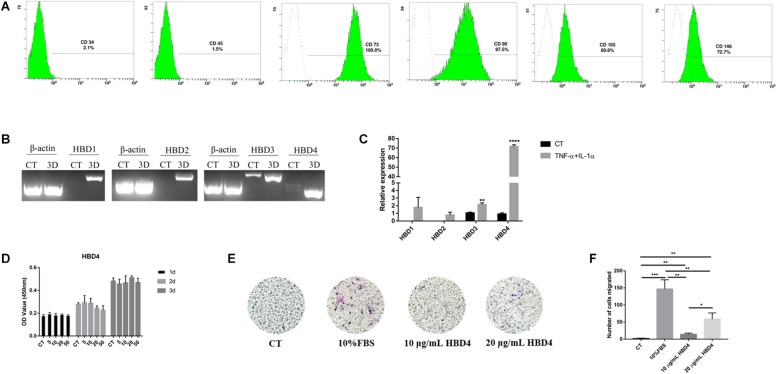
Activated expression of HBD and effects of HBD4 on biological activity of SHED. Surface marker expression of SHED **(A)**. SHED expressed low levels of CD34 (2.1%) and CD45 (1.5%), but expressed high levels of CD73 (100%), CD90 (97.5%), CD105 (80.6%), and CD146 (72.7%). The effects of proinflammatory cytokines on HBD1-4 expression in SHED were determined by qPCR and agarose gel electrophoresis **(B)**. Relative expression of HBD1-4 in CT and TNF-α + IL-1α stimulation group **(C)**. SHED were treated with different concentrations of HBD4 (0, 5, 10, 20, and 50 μg/mL) for 1, 2, and 3 days and cell viability was measured using CCK-8 assay **(D)**. Representative images of migrated cells in control, 10 μg/mL HBD4, 20 μg/mL HBD4 and 10% FBS groups **(E)**. Relative comparison of migrated cell numbers among the four groups **(F)**. The experiments were performed for three times. ^∗^*p* < 0.05; ^∗∗^*p* < 0.01; ^∗∗∗^*p* < 0.001; ^****^P < 0.0001 (CT, control; 3D, 3 days).

Mesenchymal stem cell specific markers expression was detected by flow cytometric study to identify the stemness of SHED. Cell surface marker analysis by flow cytometry showed that SHED positively expressed mesenchymal stem cell markers, including CD73, CD90, CD105, and CD146, but showed little expression of hematopoietic stem cell markers CD34 and CD45 (Figure 1A and Supplementary Data Sheet S1). The trilineage differentiation ability of SHED we used in the research was proved in our previous work (Xie et al., 2019).

The inflammatory factor combination of tumor necrosis factor alpha (TNF-α, 10 ng/mL) and interleukin (IL)-1α (10 ng/mL) resulted in HBD1-2 messenger RNA (mRNA) expression and the increased mRNA expression of HBD3–4 after 3 days of culture (Figure 1B and Supplementary Data Sheet S1). The up-regulation of HBD4 after stimulation by inflammatory factors was the most significantly compared to HBD1-3 (Figure 1C).

To determine the effect of HBD4 on viability of SHED, SHED were treated with various concentrations of HBD4 on day 1, day 2, and day 3. The viability of SHED was not affected by different concentrations of HBD4 (Figure 1D). After 24 h of migration, the group containing 10% FBS had the greatest effect on the migration of SHED, followed by 20 μg/mL HBD4 and 10 μg/mL HBD4. Compared to the control group, HBD4 groups promoted the migration of SHED, which was affected by the concentration (Figures 1E,F).

### The Ability of HBD4 to Inhibit LPS-Mediated Inflammation in SHED

**FIGURE 2:**
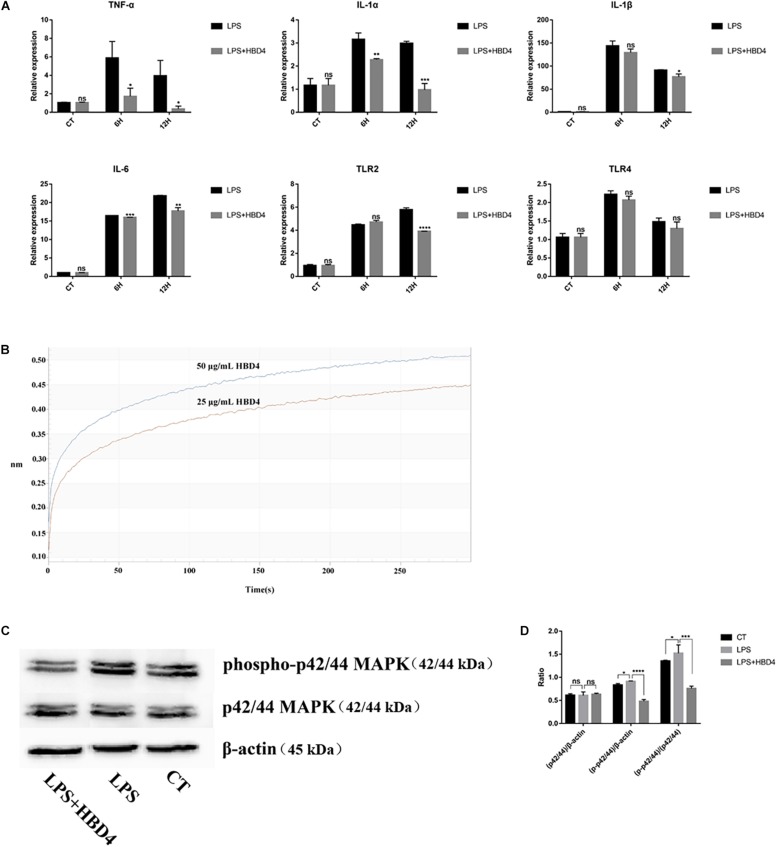
HBD4 regulated LPS-induced TNF-α, IL-6, IL-1α, IL-1β, TLR2 and TLR4 mRNA expression in SHED **(A)**. Statistical comparisons are between LPS alone versus LPS with HBD4 at the same time point in each group. The interaction between HBD4 and LPS was analyzed by Octet optical biosensors **(B)**; SHED were incubated with HBD4 and then LPS. Cellular proteins were extracted and separated by sodium dodecyl sulfate-polyacrylamide gel electrophoresis. Decreased intensity of phospho-p42/44 MAPK of HBD4 + LPS was detected in comparison with LPS alone **(C)**. The quantification of the band density was determined using Image J software **(D)**. The experiments were performed for three times. ^∗^*P* < 0.05; ^∗∗^*P* < 0.01; ^∗∗∗^*P* < 0.001; ^****^P < 0.0001; ns, no significance (CT, control; 6H, 6 h; 12H, 12 h).

SHED (5 × 10^5^ cells/mL) were pre-treated with 50 μg/mL HBD4 overnight, and then incubated with 500 ng/mL LPS. Cells collected after incubation for different time points were assayed for TNF-α, IL-6, IL-1α, IL-1β, Toll-like receptor (TLR)2, and TLR4 mRNA expression by qPCR (Figure 2A). HBD4 inhibited the LPS-mediated inflammatory response. To explore the mechanism of HBD4 inhibiting inflammatory response, we thus examined the affinity of HBD4 for LPS by the Fortebio Octet System (Figure 2B). The affinity of HBD4 to LPS increased with concentration and the high affinity of HBD4 for LPS implied that HBD4 might be involved in the anti-inflammatory effect through LPS neutralization. To validate this effect, we also examined whether HBD4 inhibited LPS-induced inflammation-related signaling pathway. We found that high level of phospho-p44/42 MAPK was reduced if SHED were preincubated with HBD4 when challenged with LPS (Figures 2C,D and Supplementary Data Sheet S2).

### *In vitro* Antibacterial Activity of HBD4 Against *F. nucleatum* and *P. gingivalis*

**FIGURE 3:**
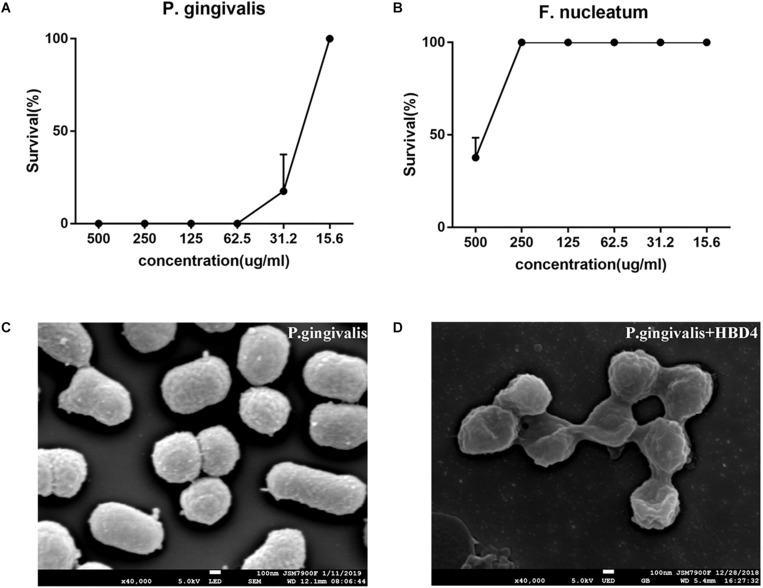
Antibacterial assay of HBD4 *in vitro* against *P. gingivalis* **(A)** and *F. nucleatum* **(B)**. The results represented the mean ± standard deviation from three independent experiments. **(C**,**D)** Showed the SEM images of the *P. gingivalis* mixed with H_2_O or HBD4, respectively. SEM × 40000.

As illustrated in Figures 3A,B and Supplementary Data Sheets S3, S4, bacterial growth was suppressed by HBD4. The minimum inhibitory concentration (MIC) of HBD4 was >250 μg/mL on 10^5^ cells/mL *F. nucleatum* and 31.2 μg/mL for *P. gingivalis*. In order to further observe the effect of HBD4 on *P. gingivalis*, we used SEM to observe the morphological changes of *P. gingivalis* after the action of HBD4. The cells of *P. gingivalis* grown in H_2_O without HBD4 appeared to be coccobacillary. The form of cells was regular and brims were slippery (Figure 3C). The cells of *P. gingivalis* grown in H_2_O containing 100 μg/mL HBD4 appeared to be widely damaged with a sunken surface, irregular form, and adhesion among cells (Figure 3D).

### The Effects of HBD4 on Osteoblast/Odontoblast Differentiation of SHED

**FIGURE 4:**
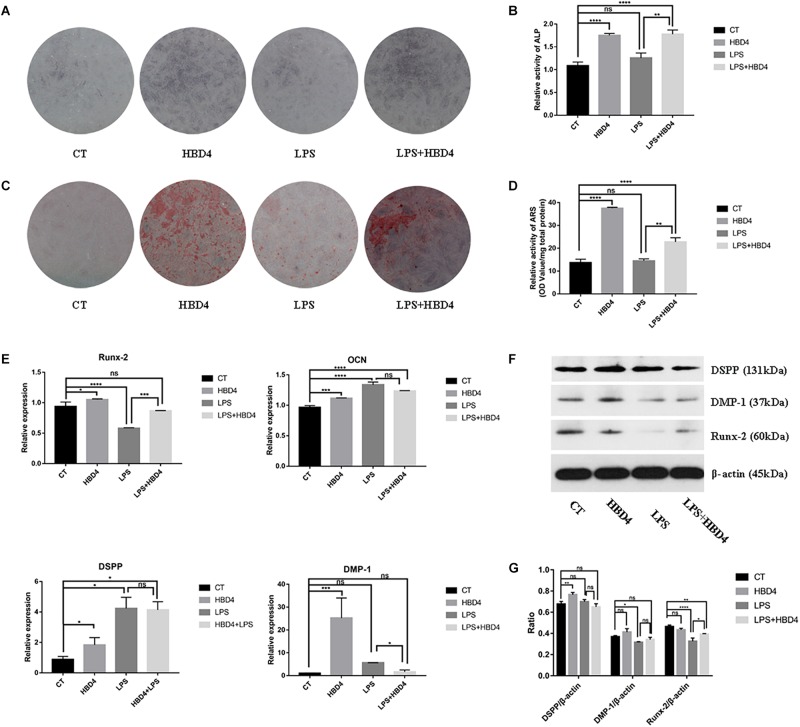
Effects of HBD4 on the osteoblast/odontoblast differentiation in SHED at 7 and 21 days. SHED were treated with HBD4 (10 μg/mL) and LPS (1 μg/mL). ALP staining at 7 days **(A,B)** and ARS staining at 21 days **(C,D)** of SHED after HBD4 and LPS treatment. Effects of HBD4 on osteoblast/odontoblast gene **(E)** and osteoblast/odontoblast protein **(F,G)** expressions of SHED in control and inflammatory microenvironments at 21 days. The experiments were performed for three times. ^∗^*P* < 0.05; ^∗∗^*P* < 0.01; ^∗∗∗^*P* < 0.001; ^****^P < 0.0001; ns, no significance (CT, control).

The effects of HBD4 with/without LPS on early osteoblast differentiation of SHED were assessed by ALP staining on 7 days (Figures 4A,B). The ALP activity of SHED treated with HBD4 was higher than that of control group. HBD4 increased the ALP activity even when SHED were stimulated by LPS. It indicated that HBD4 promoted early stage of osteoblast differentiation in SHED with/without the stimulation of LPS. The effects of HBD4 or/and LPS on late osteoblast differentiation of SHED were assessed by ARS (Figures 4C,D). The stain color was deeper in HBD4 and HBD4 + LPS group, indicating that the degree of mineralization was increased. The qPCR and western blot were applied to evaluate the mRNA and protein expression of osteoblast/odontoblast markers in SHED on 21 days. For osteoblast markers, HBD4 increased the mRNA expression of OCN without LPS and the expression of Runx-2 with LPS. For odontoblast markers, mRNA expression of DSPP and DMP-1 increased in HBD4 group versus control group (Figure 4E). Furthermore, the protein expression tendency of DSPP, DMP-1 and Runx-2 in HBD4 versus control or LPS versus LPS + HBD4 group is similar as that of mRNA expression (Figures 4F,G).

**FIGURE 5:**
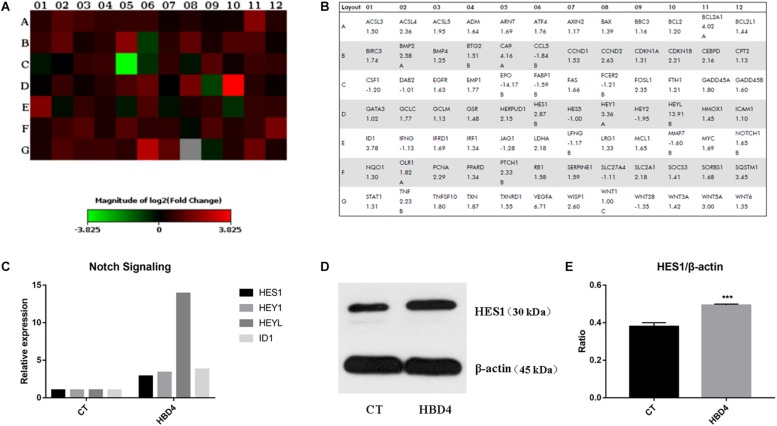
Potential signaling pathway involved in the differentiation of SHED with HBD4 for 21 days. The heat map **(A)** and table **(B)** provided a visualization of the fold changes in expression between control and HBD4 group for every gene in the array. Fold changes of molecules in Notch Signaling pathway expressed by SHED in control and HBD4 group **(C)**. Increased expression of HES1 in HBD4 group was detected by western blot in comparison with the control group **(D,E)**. PCR array data analysis was conducted at QIAGEN’S GeneGlobe Data Analysis Center and the experiments were performed for three times. ^∗∗∗^*P* < 0.001.

The Human Signal Transduction PathwayFinder RT^2^ Profiler PCR Array was applied to find the signaling pathway involved in HBD4 differentiation-promoting activity on SHED. It showed that several functional genes changed dramatically in the control group compared with the HBD4 group (Figures 5A,B and Supplementary Table S1). For Notch pathway, at day 21, HES1, HEY1, HEYL, and ID1 expression more than doubled in the HBD4 group compared with the control group (Figure 5C). Increased protein expression of HES1 in HBD4 group further indicated that Notch signaling pathway was related with HBD4 effect on osteoblast/odontoblast differentiation (Figures 5D,E).

## Discussion

In addition to efficacy comparative study of the existing pulp capping agents, there is a trend to explore new bioactive proteins for vital pulp therapy. Bioactive materials or materials containing bioactive proteins have already been applied in commercial products (da Rosa et al., 2018). As for primary teeth, to explore the pulp capping effect of active proteins in vital pulp preservation, the effects of them on biological activity of SHED should be studied first. Inspired by expression and function of HBDs in dental pulp cells, we tried to explore that of HBDs in SHED. In our study, HBD1 and HBD2 were expressed only after inflammatory stimulation. HBD3 and HBD4 were expressed in both control and stimulation groups, and HBD4 was up-regulated most significantly after TNF-α + IL-1α stimulation, which was basically consistent with the relative expression of HBDs in permanent dental pulp tissue (Paris et al., 2009). Considering the high expression and certain chemotaxis effect, we chose HBD4 for subsequent functional research in SHED.

HBDs expression can be stimulated by inflammatory factors *in vivo* and then HBDs exert the role of inflammatory immune regulation. The regulation role of HBDs is mainly manifested by inhibiting inflammation, which has been confirmed in many cell lines. HBD114, HBD123, and HBD126 can inhibit the secretion of inflammatory factors in RAW264.7 cells stimulated by LPS (Motzkus et al., 2006; Liu H. et al., 2013; Yu et al., 2013). HBD3 significantly inhibited the expression of TNF-α in RAW264.7 cells (Lyu et al., 2017), THP-1 cells (Semple et al., 2010) and human myeloid dendritic cells (Zhu et al., 2016) stimulated by LPS. However, the inflammation regulation role of HBD on SHED has not been reported. In the current study, we observed the effects of HBD4 on inflammatory factors expression in SHED stimulated by LPS. The mRNA expression levels of IL-1α, IL-6 and TNF-α were down-regulated by HBD4 at 6 and 12 h, the mRNA expression level of IL-1β was detected to decrease at 12 h. HBD4 decreased the expression of TLR2 at 12 h, but had no effect on TLR4, indicating that HBD4 regulated SHED inflammation through TLR2 rather than TLR4. In order to further elucidate the mechanism of HBD4 inhibiting LPS-mediated SHED inflammation, we observed that HBD4 had high affinity to LPS. In addition, western blot results showed that HBD4 reduced the phosphorylation of p42/44 MAPK in SHED stimulated by LPS. We speculated that HBD4 directly bond to LPS and reduced the activation of MAPK signaling pathway in SHED, thus achieving the anti-inflammatory effect. This result is consistent with the mechanism reported in the literature that members of the HBD family inhibit LPS-mediated inflammatory response (Liu H. et al., 2013).

Effective removal of bacteria is key to successful pulp capping. *F. nucleatum* and *P. gingivalis* are the most common bacteria in dental pulp infection and play a key role in the initiation stage of pulp and periapical diseases (Dahlen and Hofstad, 1977; Gomes et al., 2007). *Enterococcus faecalis* is the main pathogen of pulp reinfection, which can tolerate a harsh environment and cause persistent infection in root canals (Nakajo et al., 2006). HBD4 has a strong bactericidal effect on *E. faecalis* (Lee and Baek, 2012). However, the antimicrobial effects of HBD4 on *F. nucleatum* and *P. gingivalis* have not been reported. As HBD4 is a salt-sensitive antimicrobial agent (Sharma and Nagaraj, 2015), we use water as antimicrobial solvent in our experiments. The results showed that HBD4 had a weak antimicrobial effect on F. nucleatum and the MIC was more than 250 μg/mL. However, for 10^5^ cells/mL P. gingivalis, the MIC of HBD4 was 31.2 μg/mL. SEM images showed that the surface of *P. gingivalis* treated by HBD4 was depressed and irregular. In addition, the bacteria cells adhered to each other in contrast with the control group. HBD4 is a positively charged peptide. It may interact with negatively charged components of *P. gingivalis* cell membranes, such as lipopolysaccharide (LPS) and lead to the bacterial death. As antimicrobial ability of HBD can be improved by optimizing structures and changing net charges (Xie et al., 2005), we can also try to improve the antimicrobial ability of HBD4 against *F. nucleatum* in such ways in the future.

HBD1-3 can promote the differentiation of some stem cells and precursor cells. HBD2 and HBD3 can induce osteoblast-like cells MG63 to differentiate into osteoblasts, increase the expression level of osteoblast markers and promote mineralization (Kraus et al., 2012). The combination of HBD3 and gold nanoparticles can promote the differentiation of human periodontal ligament stem cells into osteoblasts under inflammation (Zhou J. et al., 2018). However, the differentiation induction ability of HBD4 on SHED have not yet been reported. In our study, on day 7, ALP staining in HBD4 and HBD4 + LPS groups had increased compared to the control and LPS groups, indicating that HBD4 had an effect on early-stage differentiation. On day 21, the trend in ARS results was consistent with that in ALP staining. The qPCR and western blot results of the osteoblast markers among four groups showed that HBD4 may induce the osteoblast differentiation under a normal and inflammation environment. For odontoblast markers, we found that HBD4 increased the mRNA expression of DSPP and DMP-1 in HBD4 compared with the control group, indicating that HBD4 can promote the odontoblast differentiation in SHED.

Next, we attempted to determine HBD4-related signaling pathway so that we could thoroughly understand the effect of HBD4 and control the hard tissue regeneration in future applications. The Human Signal Transduction PathwayFinder RT^2^ Profiler PCR Array results showed that during SHED differentiation induced by HBD4, Notch signaling molecule expressions changed dramatically. Notch signaling pathway plays a pivotal role in the regulation of many fundamental cellular processes and HES1 is an important target gene in Notch signaling pathway (Liu Y. et al., 2013). The protein expression of HES1 by western blot further verified the association of Notch signaling pathway. The effects of Notch signaling on osteoblast/odontoblast differentiation of stem cells may be controversial. Some studies reported that Notch signaling might inhibit stem cell differentiation (Zhang et al., 2008; Kraus et al., 2012). However, other studies found that Notch promoted osteogenic/odontogenic differentiation of mesenchymal stem cells (Cao et al., 2017; Bagheri et al., 2018; Zhou M. et al., 2018), such as SHED(Sukarawan et al., 2016). In our study, osteoblast/odontoblast differentiation induced by HBD4 was related to the activation of Notch signaling pathway.

There are still some limitations in our experiments. First, we are not able to determine the exact localization of HBD4 in SHED. We will continue to optimize the experimental conditions to figure out the immunological localization of HBD4 in SHED. Second, further *in vivo* experiments are needed to verify the actual effects of odontogenesis of HBD4 in pulp capping therapy. The study of HBD4 in SHED will help to explore new pulp capping agents for primary teeth and provide new clues for seed cells in tissue engineering of pulp regeneration.

## Data Availability Statement

The datasets generated for this study are available on request to the corresponding author.

## Ethics Statement

The studies involving human participants were reviewed and approved by the Ethics Committee of the Peking University Health Science Center (PKUSSIRB-201630091). Written informed consent to participate in this study was provided by the participants’ legal guardian/next of kin.

## Author Contributions

YeZ conceptualized, planned, and performed all the experiments. YeZ and YW wrote the manuscript. NR and JL performed the experiments of quantitative polymerase chain reaction and western blot. XL and TF performed the antibacterial experiments. YmZ and YW contributed toward editing and proofreading the manuscript. LG was responsible for the overall project design and manuscript organization, revision, and finalization.

## Conflict of Interest

The authors declare that the research was conducted in the absence of any commercial or financial relationships that could be construed as a potential conflict of interest.

## Abbreviations

HBD4: human β defensin 4
SHED: stem cells derived from human exfoliated deciduous teeth.
