# Supplementary material for: Activation and Biological Properties of Human β Defensin 4 in Stem Cells Derived From Human Exfoliated Deciduous Teeth
Source: Front Physiol. 2019 Oct 22;10:1304. doi: 10.3389/fphys.2019.01304 (PMC6817489; doi:10.3389/fphys.2019.01304)
Supplement: Supplementary file 2 [file Data_Sheet_1.ZIP › Supplementary Material 1/Primary data for SHED flow cytometry of Figure 1A/cd105.pdf]

Protocol :PE.PRO
Acq Date :31-JUL-18

Analysis Date : 08/01/18
Analysis Time : 18:52:20

(F1)[Ungated] 00011051 843.LMD : FS LIN/SS LIN

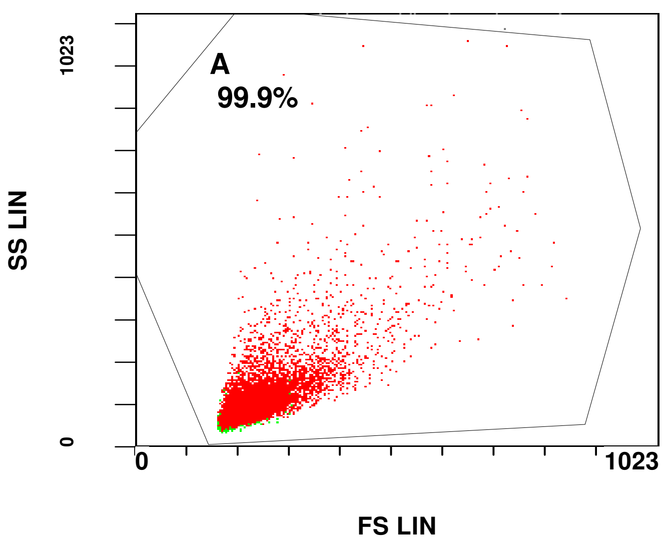

(F1)[A] 00011051 843.LMD : FL1 LOG

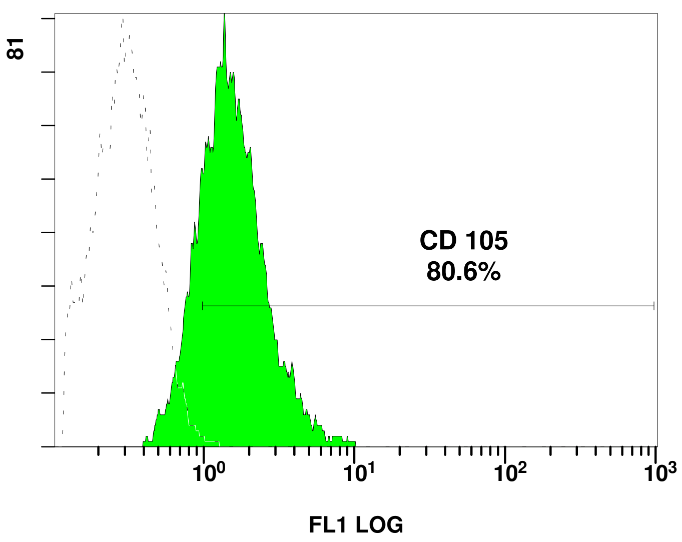

Statistical Analysis

PROGRAM INFORMATION

File:- 00011051 843.LMD				
Gate:- A				
Compensation:-				
Filename:- 00011051 843.LMD				
Mean Calculation Method:- LOG-LOG				
Region	Number	%Gated	X-Mean	X-HPCV
CD 105	8054	80.64	2.1	8.2

File:- 00011051 843.LMD				
Gate:- Ungated				
Compensation:-				
Filename:- 00011051 843.LMD				
Mean Calculation Method:- LOG-LOG				
Region	Number	%Gated	X-Mean	X-HPCV
A	9988	99.88	233.6	13.1
