# Supplementary figures and images for: Activation and Biological Properties of Human β Defensin 4 in Stem Cells Derived From Human Exfoliated Deciduous Teeth
Source: Front Physiol. 2019 Oct 22;10:1304. doi: 10.3389/fphys.2019.01304 (PMC6817489; doi:10.3389/fphys.2019.01304)

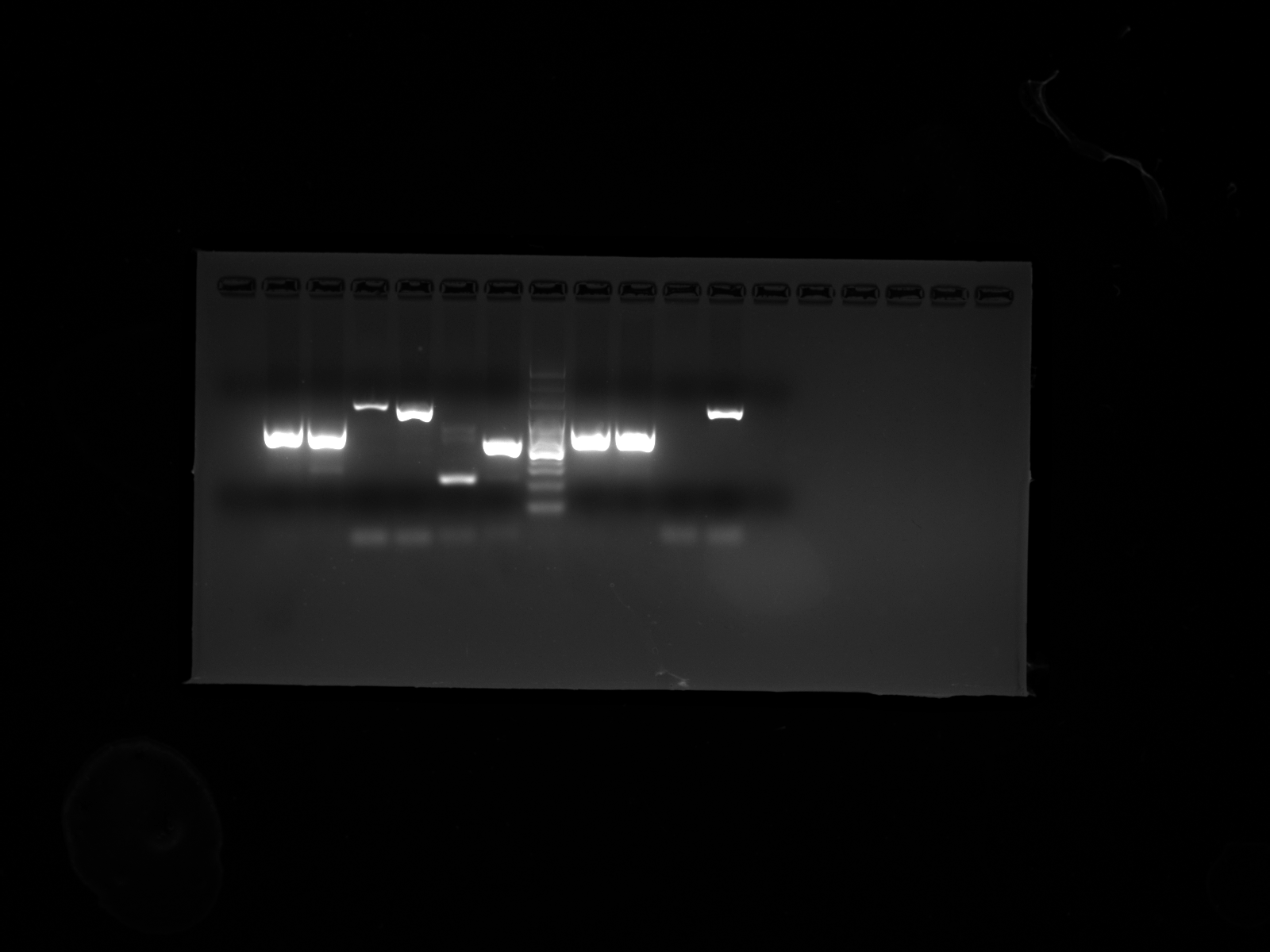

Supplement: Supplementary file 2 [file Data_Sheet_1.ZIP › Supplementary Material 1/Agarose gel electrophoresis picture of Figure 1B/HBD1,HB3,HBD4.Tif]

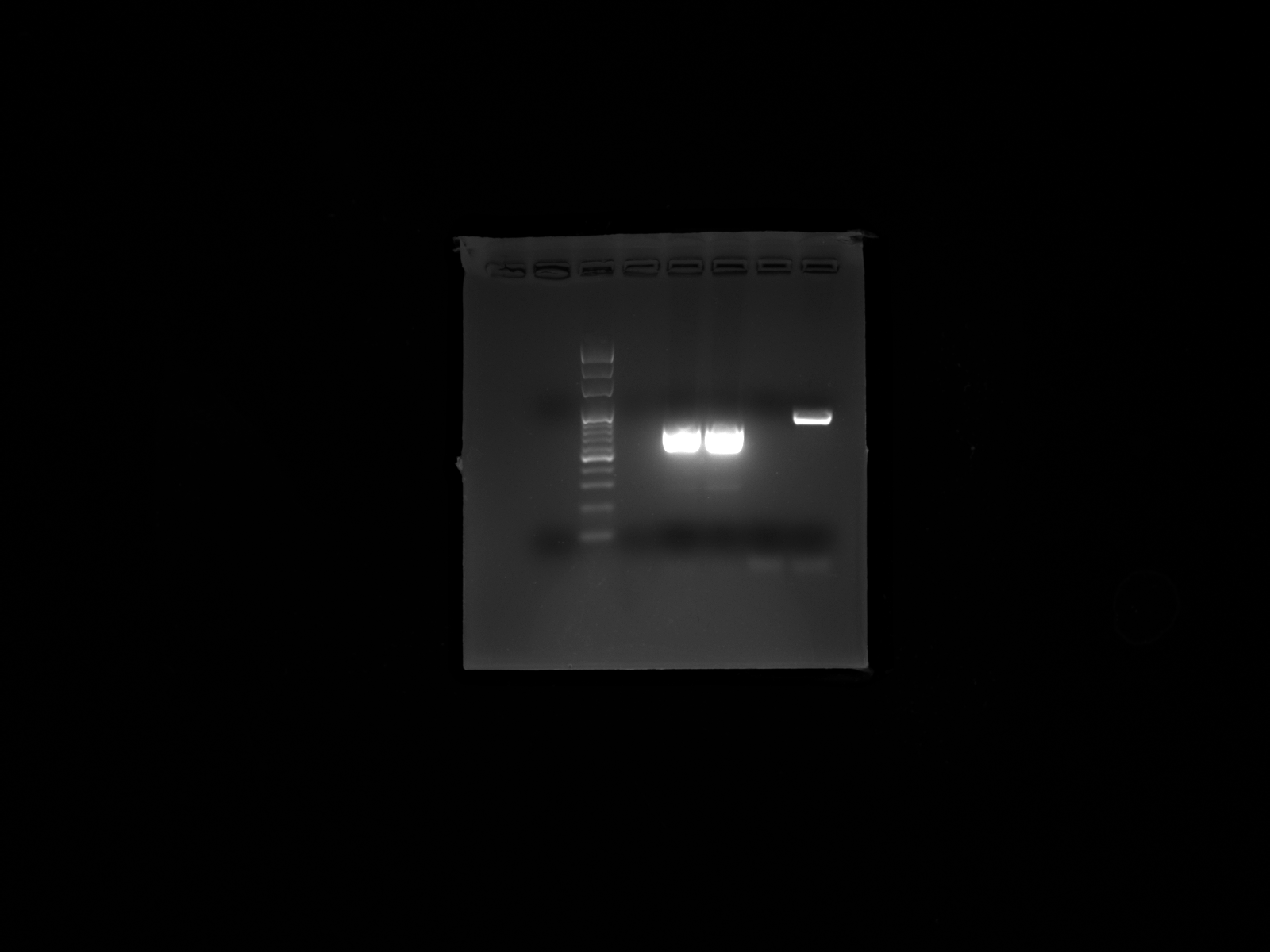

Supplement: Supplementary file 2 [file Data_Sheet_1.ZIP › Supplementary Material 1/Agarose gel electrophoresis picture of Figure 1B/HBD2.Tif]

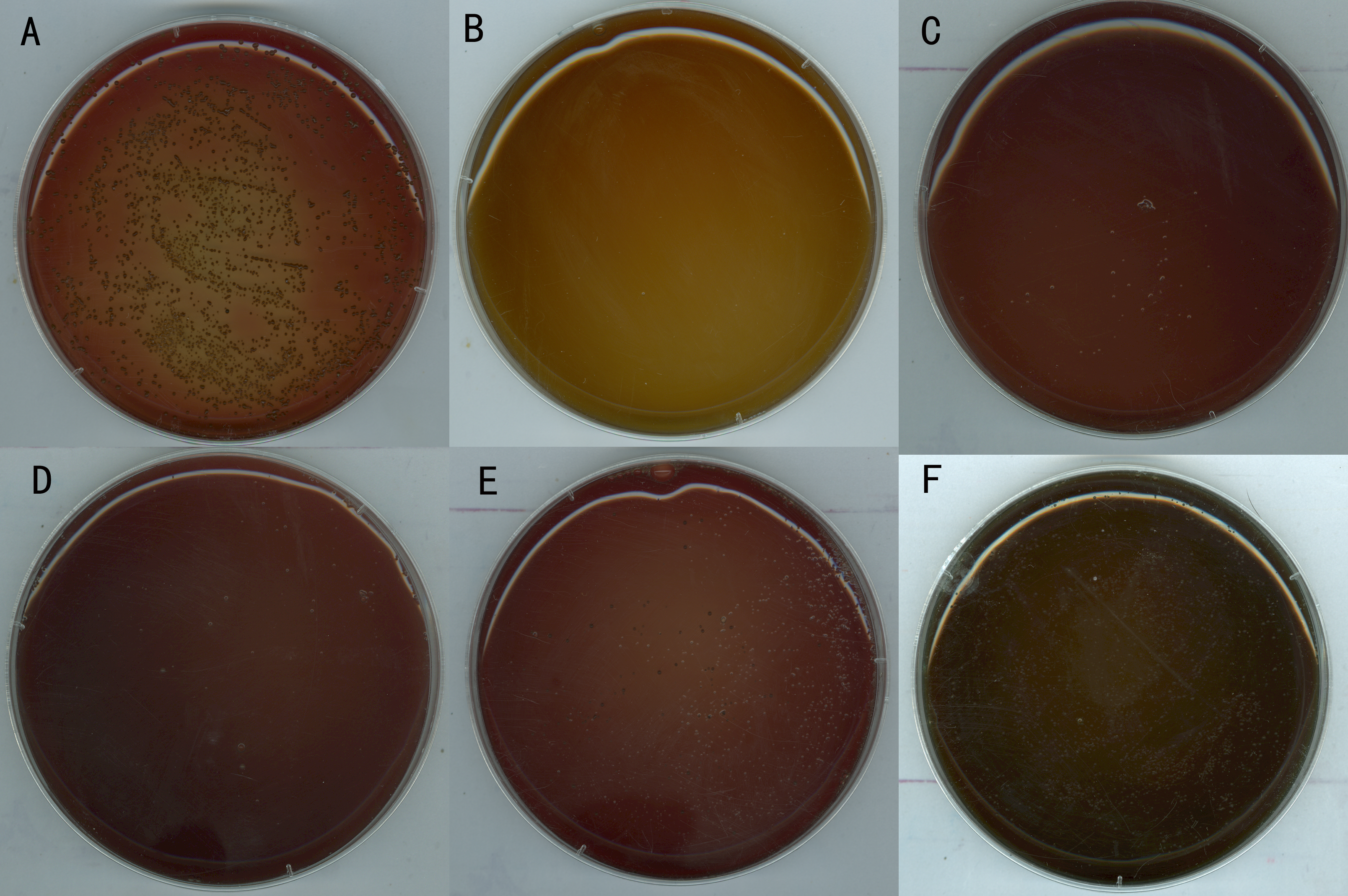

Supplement: Supplementary file 4 [file Data_Sheet_3.ZIP › Supplementary Material 3/Antibacterial blood plate photographs of Figure 3A/PG.tif]

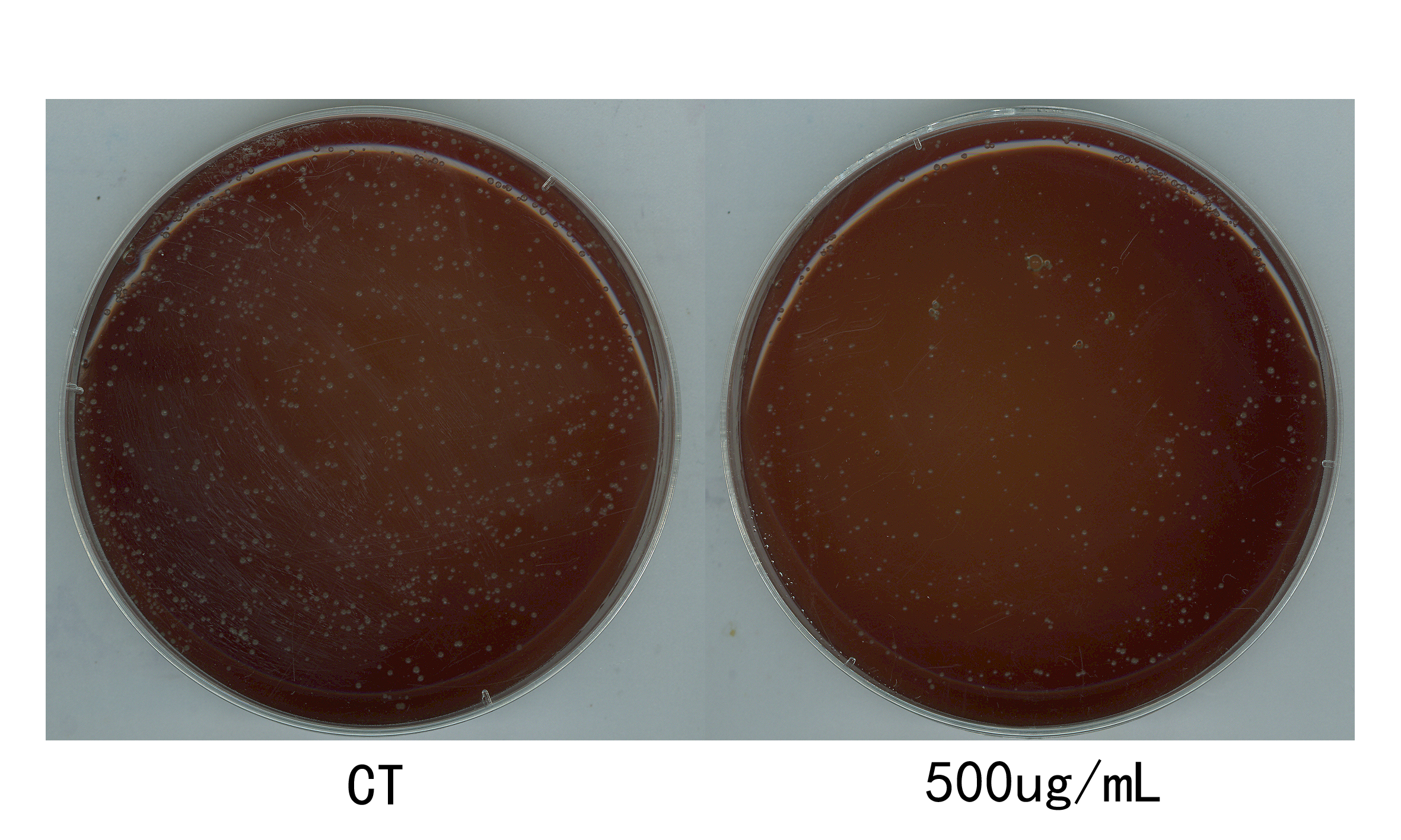

Supplement: Supplementary file 5 [file Data_Sheet_4.ZIP › Supplementary Material 4/Antibacterial blood plate photographs of Figure 3B/FN.tif]
